# Endothelial dysfunction and platelet hyperactivity in type 2 diabetes mellitus: molecular insights and therapeutic strategies

**DOI:** 10.1186/s12933-018-0763-3

**Published:** 2018-08-31

**Authors:** Raminderjit Kaur, Manpreet Kaur, Jatinder Singh

**Affiliations:** 1 Department of Molecular Biology & Biochemistry, Guru Nanak Dev University, Amritsar, Punjab India; 2 Department of Human Genetics, Guru Nanak Dev University, Amritsar, Punjab India

**Keywords:** Hyperglycemia, Insulin resistance, Inflammation, Oxidative stress, Vascular complications

## Abstract

The incidence and prevalence of diabetes mellitus is rapidly increasing worldwide at an alarming rate. Type 2 diabetes mellitus (T2DM) is the most prevalent form of diabetes, accounting for approximately 90–95% of the total diabetes cases worldwide. Besides affecting the ability of body to use glucose, it is associated with micro-vascular and macro-vascular complications. Augmented atherosclerosis is documented to be the key factor leading to vascular complications in T2DM patients. The metabolic milieu of T2DM, including insulin resistance, hyperglycemia and release of excess free fatty acids, along with other metabolic abnormalities affects vascular wall by a series of events including endothelial dysfunction, platelet hyperactivity, oxidative stress and low-grade inflammation. Activation of these events further enhances vasoconstriction and promotes thrombus formation, ultimately resulting in the development of atherosclerosis. All these evidences are supported by the clinical trials reporting the importance of endothelial dysfunction and platelet hyperactivity in the pathogenesis of atherosclerotic vascular complications. In this review, an attempt has been made to comprehensively compile updated information available in context of endothelial and platelet dysfunction in T2DM.

## Background

Diabetes mellitus (DM) is a chronic hyperglycemic disease condition attributed to defective insulin secretion or action or both [1]. According to World Health Organization (WHO), 1.5 million people died with DM in 2012. Moreover, more than 80% of the total deaths occurred due to DM are from low- and middle-income countries [2]. The prevalence of diabetes is also increasing very rapidly in India. According to International Diabetes Federation (IDF) (2017), 72 million cases of diabetes were reported from India with a prevalence of 8.8% [3]. Most of the cases with DM either have Insulin dependent DM (known as type 1 DM) or non-insulin dependent DM (known as type 2 DM).

T2DM, also known as “adult-onset diabetes,” is the most prevalent form of diabetes and accounts for approximately 90–95% of the total diabetes cases worldwide. T2DM is characterized by progressive insulin deficiency and impairment of β-cell function, superimposed on insulin resistance. It is a multisystem disease associated with both micro-vascular and macro-vascular complications. The micro-vascular complications include diabetic retinopathy, neuropathy and nephropathy [4]. The macro-vascular complications are manifested as accelerated atherosclerosis that results into severe peripheral vascular disease, premature coronary artery disease (CAD) and increased risk of cerebrovascular diseases [5–8]. Approximately 80% of the diabetic mortality is attributed to the thrombotic events, out of which 75–80% deaths are caused by cardiovascular complications. Moreover, patients with T2DM have two to four fold higher risk of recurrent atherothrombotic events and vascular complications as compared to non-DM patients [9, 10].

**Fig. 1:**
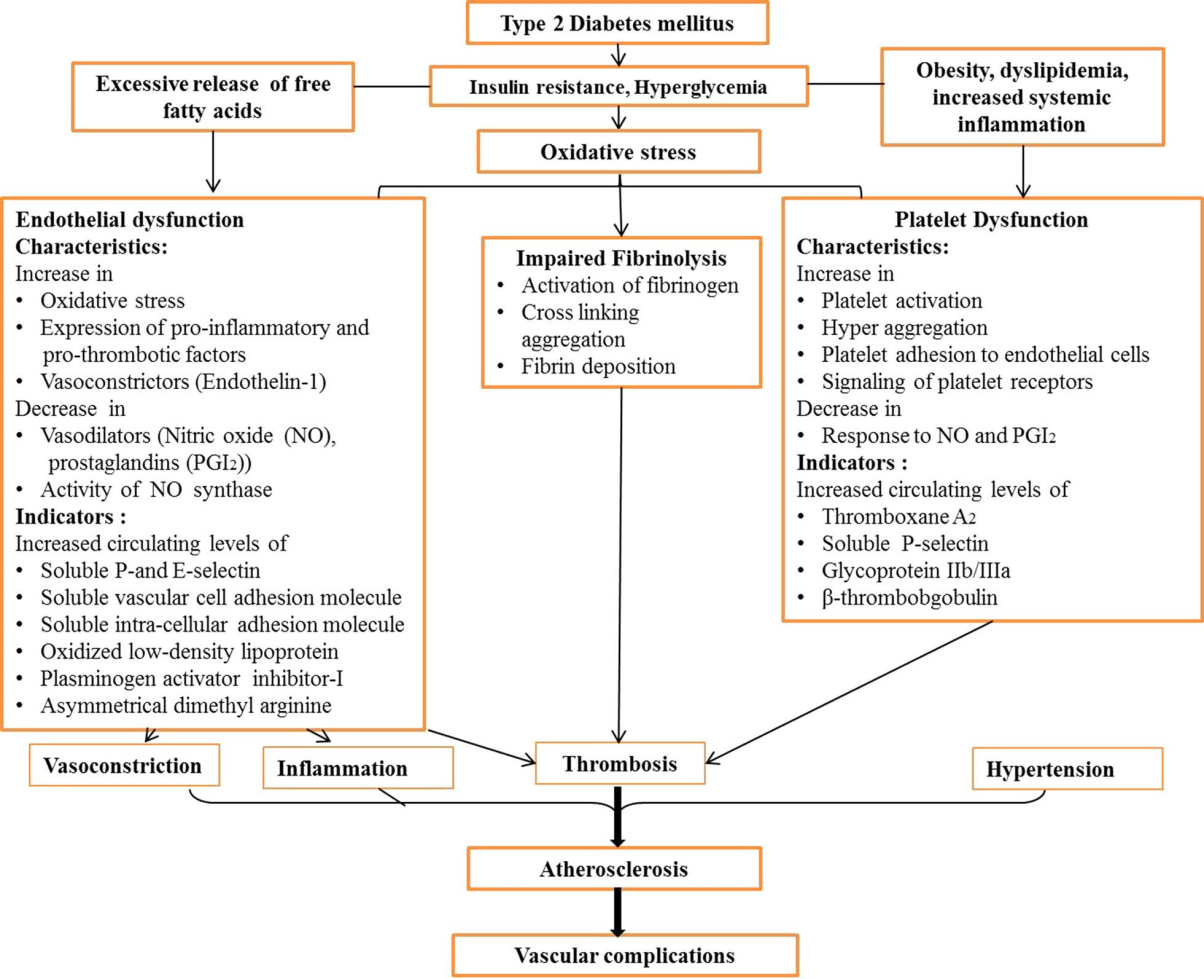
Pathophysiological events leading to vascular complications in T2DM patients

The metabolic milieu of T2DM, including insulin resistance, hyperglycemia and release of excess free fatty acids, along with other metabolic abnormalities affects vascular wall by a series of events including endothelial dysfunction, platelet hyperreactivity, oxidative stress and low-grade inflammation. Activation of these events further enhances vasoconstriction and promotes thrombus formation, ultimately resulting in the development of atherosclerosis (Fig. 1) [6, 8]. The endothelial dysfunction is the key event that initiates the inflammatory mechanisms associated with vascular complications in T2DM patients [11]. Furthermore, it is an initial event of atherogenesis, which involves imbalance in the tightly regulated equilibrium of vasodilators and vasoconstrictors together with the inhibition of the anticlotting systems. These changes further impair vasorelaxation and increase proliferation of vascular smooth muscle cells [12]. Along with other contributors of prothrombotic state in T2DM, platelet dysfunction plays a pivotal role [13]. Activation of endothelium by increased release of cytokines and expression of adhesion molecules mediates platelet activation and adhesion to activated endothelium [13–16]. Impaired endogenous inhibition of platelets makes platelets more susceptible to activation and activated endothelium provides more adhesion molecules and platelet agonists. Activated platelets then mediate leukocyte recruitment, which in turn activated by subsequent interaction. Subsequently, after firm adhesion to activated endothelium, leukocyte transmigrates and presents to lipid and cells in vessel wall. This accelerates the further inflammatory reactions in the vascular wall and promotes atherosclerotic vascular complications [13–16]. Therefore, understanding of mechanism of endothelial and platelet dysfunction, unquestionably involved in pathogenesis of T2DM, is required to ameliorate the adverse vascular events, leading to prothrombotic state in T2DM. An attempt has been made to comprehensively compile updated information available in context of endothelial and platelet dysfunction in this chronic disease condition.

## Endothelial dysfunction in T2DM

The endothelium comprises a single cell layer lining the inner surface of vascular lumen and act as a barrier between blood and vessel wall [17]. The endothelial cells perform multiple functions including regulation of cell adhesion, tissue growth and metabolism, angiogenesis, inflammatory responses, vessel integrity, hemostasis, vascular permeability, vascular smooth muscle cell proliferation, platelet activation, fibrinolysis, thrombus formation and maintaining blood fluidity [18–21]. Endothelium is also responsible for maintaining vascular tone by production of vasodilators including nitric oxide (NO) and prostacyclin (PGI_2_), and vasoconstrictors like endothelin-1 (ET-1), angiotensin II and reactive oxidative species (ROS) [22–24]. Endothelial cells also release prothrombotic molecules including von Willebrand factor (vWV) (promotes platelet aggregation), plasminogen activator inhibitor-1 (PAI-1) (inhibits fibrinolysis) and antithrombotic molecules including NO and PGI_2_ (inhibit platelet aggregation) [19].

### Hyperglycemia and endothelial dysfunction

**Fig. 2:**
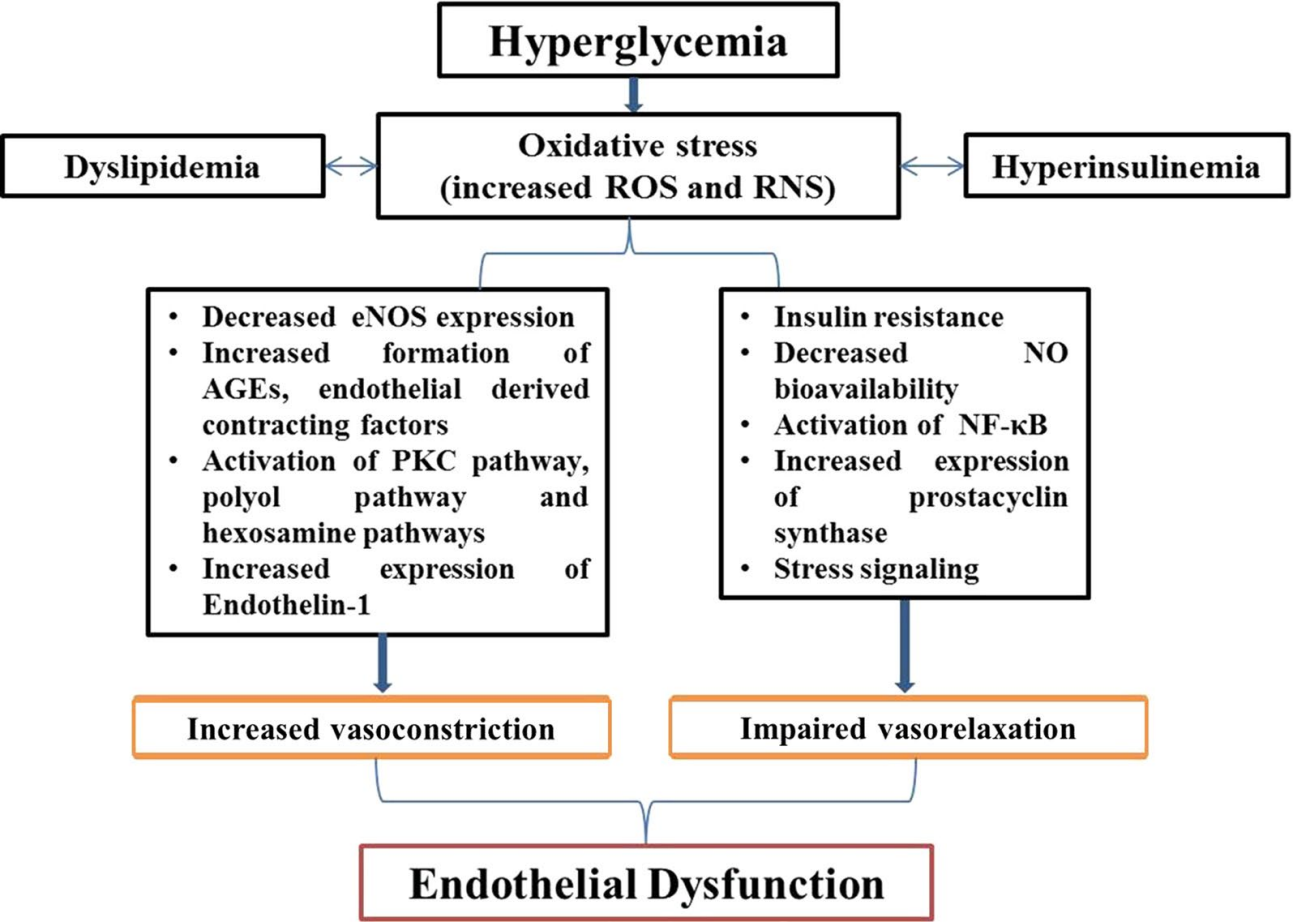
Endothelial dysfunction in diabetes: Hyperglycemia leads to increase production of ROS and RNS resulting oxidative stress. Oxidative stress affects vascular homeostasis by causing increased vasoconstriction and impaired vasorelaxation and eventually leads to endothelial dysfunction. *ROS* reactive oxygen species, *RNS* reactive nitrogen species, *eNOS* endothelial nitric oxide synthase, *NO* nitric oxide, *AGE* advanced glycation end products, *PKC* protein kinase, *NF-κB* nuclear factor-κB

Studies have shown that impaired endothelial function in both macro- and micro-vascular complications owing to prolonged, transient and acute hyperglycemia in animal models as well as human participants [25–28]. Although, effects of intensive glycemic control were well profound for prevention of micro-vascular complications as compared to macro-vascular disease reduction [29]. Hyperglycemia is thought to trigger vascular damage by creating imbalance between NO bioavailability and accumulation of ROS as well as reactive nitrogen species (RNS, resulting in endothelial dysfunction) (Fig. 1). Furthermore, hyperglycemia damage the vascular bed by several cellular mechanisms, comprising of enhanced production of intracellular advanced glycation end products (AGEs), increased expression of AGE receptors (RAGE) and ligands, augmented polyol and hexosamine flux, activation of protein kinase C (PKC) and over activation of hexosamine pathway [30]. The main underlying pathway is oxidative stress. Furthermore, in vivo and in vitro studies have shown that chronic levels of oxidative stress are among the earliest abnormalities in the natural history of insulin resistance and T2DM [31–34]. However, there is scarcity of data regarding oxidative stress in T2DM in human participants [35–38]. In a recent study, biomarkers of oxidative stress i.e. oxidized LDL and F2-isoprostanes, have been shown to be positively correlated with incident T2DM, whereas this association was attenuated with adjustment for BMI [39]. Increased ROS impairs glucose metabolism by glycolysis and increase its flux through alternative polyol and hexosamine pathways [40]. Furthermore, hyperglycemia mediated oxidative stress induces DNA damage and production of ADP-ribose polymer by activation of nuclear poly (ADP-ribose) polymerase (PARP) and reduction of glyceraldehyde-3-phosphate dehydrogenase activity. This eventually increases the levels of all the upstream glycolytic intermediates that trigger above mentioned various damaging mechanisms. The overall consequences of these mechanisms are increased vascular permeability, oxidative stress and apoptosis. Hyperglycemia also activates nuclear factor-κB (NF-κB) that mediates low-grade vascular inflammation [7, 30, 41]. Furthermore, NF-κB activation leads to augmented production of vascular adhesion molecules, cytokines and chemo-attractants resulting in activation of inflammatory cells in vascular wall. Additionally, glucotoxicity leads to increased expression of coagulant tissue factors like PAI-1, resulting in a prothrombotic state. Along with hyperglycemia, dyslipidemia and hyperinsulinemia also affect vascular tone through imbalance of vascular tone regulators [42] (Fig. 2).

AGEs formation further lead to alteration of structural, functional and receptor recognition properties of matrix components. Sequentially, AGEs binding to its receptor (RAGE) increases superoxide production that promotes macrophage induced vascular inflammation [43–45]. Furthermore, AGEs also induces decreased endothelial NO synthase (eNOS) expression as well as NO synthesis and increased ET-1 expression, leading to endothelial dysfunction [46–48]. Thus, under hyperglycemic conditions, altered formation of various biochemicals including AGEs, ROS, RNS, 3-deoxyglucosone, Amadori products, diacylglycerol and methylglyoxal, significantly contributes to endothelial dysfunction in diabetic patients [49].

An association was observed between good glycemic control and improved microvascular function in recently diagnosed T2DM patients with CVD [50]. However, in patients with prolonged disease duration, this association was lost. Furthermore, Dextrose-mediated hyperglycemia led to impaired endothelial cell proliferation as well as migration in Human umbilical vein endothelial cells by inhibiting the activation of ERK, p38 and Akt pathways [51]. These events were mitigated by co-incubation with HDL, indicating the protective effect of HDL in impaired glucose homeostasis [51]. Furthermore, HDL has also shown to have anti-inflammatory properties through suppression of pro-inflammatory cytokines and cell adhesion molecules, which can reduce endothelial activation. However, anti-inflammatory capacity of HDL was shown to be markedly impaired in T2DM patients, which can be partially attributed to chronic hyperglycemia, persistent low grade inflammation and reduced serum paraoxonase/arylesterase 1 (PON-1) activity [52]. This decrease in anti-inflammatory protection capacity of HDL leads to increased atherosclerosis risk in T2DM patients [53]. Furthermore, glucose fluctuation were also reported to contribute to chromatin remodeling and may describe the persistent vascular dysfunction in uncontrolled T2DM (HbA1c > 7.5% [54]. In addition, microRNA, miR-29, is reported to play an important role in maintaining normal endothelial function by inducing NO production. miR-29 expression has been shown to be deregulated in T2DM patients [55]. Intraluminal delivery of miR-29a-3p or miR-29b-3p mimics restored normal endothelium-dependent vasodilation (EDVD) in these patients, which otherwise showing impaired EDVD. These evidences suggested the therapeutic potential of miR-29 against cardiometabolic disorders.

### Insulin resistance and endothelial dysfunction

Insulin resistance, a key feature of T2DM, is characterized by the reduced ability of insulin to promote glucose uptake in multiple organs including skeletal muscle, adipose tissue and heart, and to restrain hepatic glucose output [55, 56]. Insulin resistance often preceded onset of hyperglycemia and diabetes for many years [57]. Obesity is a pivotal factor in increasing incidence and prevalence of insulin resistance and its vascular complications [58]. Insulin signaling involves two major pathways i.e. Phosphatidylinositol-3-kinase (PI3K) dependent pathway responsible for metabolic and hemodynamic effects and mitogen activated protein kinase (MAPK)-dependent pathway for regulation of gene expression, differentiation and cell growth. Generally, insulin promotes NO production by activating eNOS through PI3K-dependent pathway and ET-1 secretion via MAPK-dependent pathway [59, 60]. Under the conditions of insulin resistance, PI3K pathway is impaired, resulting in decreased NO production and MAPK pathway is activates, leading to increased production of ET-1, ultimately resulting in endothelial dysfunction [61]. Insulin resistance also increases expression of PAI-1, a coagulant tissue factor and adhesion molecules [62].

Additionally, insulin resistance stimulates the proliferation of vascular smooth muscle cells (VSMCs) and excessive release of free fatty acids (FFAs) in adipose tissue, which subsequently increase oxidative stress and PKC activation [63]. Insulin resistance induced excessive release of FFAs is further involved in the development of pro-atherogenic lipid profile and dyslipidemia. Eventually, increased serum levels of PAI-1, ET- 1, tumor necrosis factor-α (TNF-α), interleukin-6 (IL-6), and C-reactive protein (CRP), reflect association of insulin resistance with low-grade inflammation and endothelial dysfunction [63, 64].

In past years, the usefulness of Homeostasis model assessment of insulin resistance (HOMA-IR), as an index of insulin resistance in T2DM patients, has become a focus of much attention [65–67]. In addition, insulin resistance assessed by HOMA-IR, in non-diabetic patients with chest pain and without myocardial perfusion defects, was also shown to be associated with endothelial dysfunction, conferring independent prognostic information [68]. Furthermore, insulin resistance adipocyte-derived exosomes (IRADEs) have been shown to promote plaque vulnerability and plaque burden, partly through sonic hedgehog (shh), by mediating vasa vasorum angiogenesis in diabetic ApoE−/−mice. These effects were attenuated by silencing of shh gene and this approach has been suggested as a novel therapeutic strategy in treatment of diabetic atherosclerosis [69].

### Excessive free fatty acids and endothelial dysfunction

Increased circulating levels of FFAs are attributed to excessive release from adipose tissue and reduced uptake by skeletal muscles [70, 71]. In vivo as well as studies including human participants have suggested the acute infusion of FFAs diminishes endothelium-dependent vasodilation [7, 72]. Excessive FFAs may cause lipotoxicity that impair normal endothelial function by same mechanisms and to the same extent as by glucotoxicity. FFAs induce vasculature ROS production by increasing the expression and protein content of NADPH oxidases, and via mitochondrial uncoupling [30, 63]. It also stimulates excessive superoxide production, leading to inactivation of important anti-atherogenic enzymes i.e. eNOS and PGI_2_ synthase. FFA-induced ROS production resulted into decreased concentration of intracellular glutathione, making vasculature more susceptible to oxidative damage. It also activates NF-κB, leading to the activation of inflammatory cascade [73–75]. FFAs activate IKKα, which impairs insulin-induced production of eNOS and NO in endothelial cells [76]. Furthermore, FFAs also activates PKC that leads to increased serine phosphorylation of insulin resistance substrate (IRS)-1, resulting into decreased activation of phosphoinositide-dependent kinase-1, PI-3K, Akt, and eNOS, and ends up with impaired endothelium NO production [77, 78]. Eventually, increased FFAs lead to decreased NO bioavailability, increased vascular oxidative stress, endothelial apoptosis and augmented inflammation [79]. In diabetes, insulin resistant visceral adipocytes augment influx of FFA in arterial endothelial cells that further activate metabolite sensitive pathways of vascular damage. This may explain a link between insulin resistance and macrovascular diseases [30, 80]. Furthermore, insulin resistance increase oxidation of fatty acids, which leads to augmented oxidative stress in diabetic macrovasculature, while increased hyperglycemia-derived ROS production in diabetic microvascular diseases. Thus, oxidative stress seems to be a common pathway for triggering vascular dysfunction in both types of diabetic vascular complications [81, 82].

### Assessment and biomarkers of endothelial dysfunction in T2DM

Generally, endothelial dysfunction is evaluated by assessing NO-dependent endothelium relaxation i.e. vasodilation. In macrocirculation, endothelial function can be estimated by measuring coronary artery diameter after intra coronary infusion of agonists (e.g. acetylcholine) by quantitative angiography and at microcirculation level by assessing change in blood flow through intravascular ultrasound [83, 84]. This method is regarded as “gold standard” technique for assessment of endothelial function and has the highest clinical value, since it is used to assess the vascular bed involving atherosclerosis process and cardiac events. As forearm blood flow is invasive, another method was introduced in past decades that involve flow-mediated dilatation (FMD) of the brachial artery in response to shear stress. In this technique, changes in arterial diameter are measured by high resolution doppler ultrasonography. Escalating and deflating a blood pressure cuff was used to induce blood flow in brachial artery by shear stress (reactive hyperemia). Peripheral endothelial function is assessed using finger plethysmography by following same principle. Another technique, peripheral arterial tonometry (EndoPAT) is used to record increased digital pulse amplitude in response to reactive hyperemia and it follows the same principle of endothelial-induced vasodilatory response to acetylcholine [18, 85]. Furthermore, various agents/intervention induced capillary blood flow in several vascular beds, increasing endothelial-mediated blood flow is monitored by positron emission tomography (PET) [86–88].

Moreover, increased levels of biomarkers of inflammation, oxidative stress and hemostasis including levels of soluble inter-cellular adhesion molecule (sICAM-1), soluble vascular cell adhesion molecule (sVCAM), soluble P-selectin (sP-selectin), soluble E-selectin (sE-selectin), asymmetrical dimethylarginine (ADMA), oxidized low-density lipoprotein (oxLDL), PAI-1, vWF and CRP are used as important indicators for endothelial function [11, 84, 89]. Furthermore, overexpression of ET-1 was reported to exaggerate diabetes-induced endothelial dysfunction by altering oxidative stress [90]. Moreover, interaction between TNF-α and IL-6 also exacerbate oxidative stress and reduce phosphorylation of eNOS, contributing to increase endothelial dysfunction in T2DM patients [91].

### Pharmacological influences on endothelial function and its biomarkers

Recent studies have recognized signaling of wingless-type family member (Wnt) 5a through c-jun N-terminal kinase (JNK) as a metabolic dysfunction regulator with potential significance to vascular function [92, 93]. Furthermore, it was observed that noncanonical Wnt5a signaling and JNK activity may contribute to vascular insulin resistance as well as endothelial dysfunction and may represent a novel therapeutic target to protect the vasculature in patients with diabetes mellitus [94]. Furthermore, endothelium possess a limited inherent self-repair ability because of being formed from terminally differentiated cells of low proliferative potential, called as endothelial progenitor cells (EPCs). Previous studies have stated the prime role of EPCs in progression and development of vascular complications in T2DM patients [95, 96]. Another study has demonstrated effects of chronic administration of phosphodiesterase inhibitors on endothelial markers in T2DM patients [97]. In this study, beneficial effects of chronic use of PDE5i on endothelial function were observed. Furthermore, chronic administration of Sildenafil was found to improve serum pro-inflammatory maker (IL-6) and hemodynamic parameter (FMD) T2DM patients. Moreover, another study has reported the role of impaired autophagy in endothelial dysfunction in diabetic patients, which may be considered as a therapeutic target for diabetic vascular compilations [98]. In endothelial cells, eNOS uses l-arginine to produce NO, where arginase utilizes l-arginase to produce ornithine and urea [99, 100]. In addition, arginase up-regulation has been shown to reduce NO production by reducing bioavailability of l-arginine to eNOS, thus playing an important role in vascular dysfunction in diabetes [101]. Furthermore, endothelial function was reported to be improved in T2DM patients, without changes in HbA1c levels, by intervention with a fiber-rich diet with brown rice possibly through reduction of postprandial glucose excursions [102]. Furthermore, activation of nuclear factor (erythroid‑derived 2)‑like 2 (Nrf2), a ubiquitously expressed redox sensitive transcription factor, has been shown to attenuate endothelial dysfunction, along with downregulation of inflammatory as well as pro-oxidant genes and reduction of leukocyte–endothelial interactions [103–107]. This represents a novel therapeutic approach to inhibit diabetes related vascular injury. Furthermore, a study including diabetic patients has suggested that arginase inhibitors may prevent the endothelial dysfunction and maintain NO levels in these patients [108]. Moreover, Ipragliflozin (a novel selective sodium–glucose cotransporter 2 (SGLT2) Inhibitor) has been shown to improve hyperglycemia and prevent the development of endothelial dysfunction in a streptozotocin-induced diabetic mouse [109]. Furthermore, another SGLT2 Inhibitor, dapagliflozin have shown to reduce blood pressure and improve glycemic control in T2DM patients [110–112]. In addition to this, acute administration of dapagliflozin resulted into significantly improved systemic endothelial function, renal resistive index, arterial stiffness and parameters associated with the early stages of vascular remodeling [110, 113]. Moreover, as add-on therapy to metformin for 16 weeks, dapagliflozin improves FMD assessed endothelial function in patients with inadequately controlled early-stage T2DM [114]. Additionally, dipeptidyl peptidase inhibitors (such as linagliptin and voglibose), one of the recently introduced classes of oral glucose-lowering drugs, has been shown to significantly improve microvascular function in the fasting state and ameliorate cardiometabolic and renal parameters in the newly diagnosed T2DM and CAD patients [115, 116]. Furthermore, various cross-sectional studies have observed elevated levels of ADMA in T2DM Patients with macrovascular diseases [117, 118]. Moreover, ADMA is suggested as an independent risk factor for mortality and CVD in a wide spectrum of populations [119, 120]. As mentioned previously, accelerated atherosclerosis in T2DM is also associated with decreased activity of PON-1. Rosuvastatin, a class of statins, was shown to improve microvascular reactivity with associated beneficial changes in the postprandial levels of ADMA and PON-1 [121]. Furthermore, a continuous intravenous infusion of glucagon-like peptide-1 (GLP-1) analogs was reported to improve the blood glucose-independent vascular endothelial dysfunction in T2DM patients with stable CAD [122]. Moreover, exenatide (GLP-1 analog) was shown to inhibit the postprandial vascular endothelial dysfunction and was suggested to have multiphasic anti-atherogenic actions involving not only glucose but also lipid metabolism [123]. It was also reported that GLP-1 receptors are expressed on vascular endothelial cells and directly increase the production of NO and restrict the expression of endothelial cell adhesion factors [124, 125]. Furthermore, Biomarkers of inflammation and endothelial dysfunction such as ICAM-1 and E-selectin were observed to be positively correlated with incident T2DM, adding to the estimation of T2DM beyond a common risk score [126]. Additionally, circulating microparticles (endothelial- and platelet derived) levels have been proposed to be one of the important procoagulant determinants in T2DM patients [127]. Levels of endothelial- derived microparticles such as CD105^+^, PECAM-1, 62E^+^ and CD106^+^ as well as platelet-derived microparticles including P-selectin, fibrinogen, TF have been reported to be significantly higher in T2DM patients as compared to healthy controls [79, 128–130]. Furthermore, miRs (miR-320, miR-221, miR-222, miR-503, miR-126), a class of small non-coding RNAs, are suggested to play an important role in pathogenesis of vascular damage induced by hyperglycemia [131, 132]. Various microarray studies have observed the altered expression of miRs in T2DM patients [132–134]. A recent study has shown the critical involvement of miR-503 in hyperglycemia-induced endothelial dysfunction in diabetic mice and up-regulation of ischemic limb muscles in diabetic subjects. Remarkably, inhibition of miR-503 resulted in normalization of post-ischemic neovascularization and blood flow recovery in these diabetic mice [135]. Furthermore, numerous studies have shown decreased plasma concentration of adiponectin (APN), a multifunctional adipocytokine of adipose tissue, in T2DM patients, indicting its important role in pathogenesis of T2DM [136–138]. Additionally, T-cadherin (T-cad), a unique member of the cadherin family, has been identified as an important receptor of APN [139–142]. A recent in vivo study has shown that T-cad deficiency may cause endothelial dysfunction in T2DM vascular segments, suggesting an important role of T-cad in pathogenesis of T2DM. Therefore, altering T-cad deficiency may represent another potential therapeutic strategy in the amelioration as well as prevention of vascular injury in the T2DM [143].

## Platelets hyperactivity in T2DM

Platelets were first discovered in 1882 by Giulio Bizzozer and the multifunctional attributes of platelets were remained source of interest for biologists for many years [144]. Platelets are mainly involved in hemostasis by initiating the blood coagulation mechanism. Preferably, these remain in inactive state and only get activated by a vascular injury. On activation, platelets release several prothrombotic components from their granules through aggregation. These components including coagulation factor V, fibrinogen and vWV. Activated platelets showed altered expression of various surface glycoproteins (GP). In this activation phase, P-selectin, an adhesion molecule, stored in Weibel Palade bodies of endothelial cells and α granules of platelets is translocated to the surface [145]. GPIIb–IIIa, present on surface of activated platelets, undergoes conformational changes by activation and binds to fibrinogen. In these thrombin-activated platelets, binding of GPIV to thrombospondin (GP with antiangiogenic function) is increased, while binding sites of the vWF on GPIb-IX complex gets down-regulated [146].

To prevent thrombus formation, endothelium regulate the activity of proaggregants (such as thrombin, collagen, ADP and TxA_2_) and releases some antiaggregants, such as NO and PGI_2_ in normal blood vessels [147]. In contrast to proaggregants which show their effects after binding to specific platelet surface receptor, NO crosses the membrane and induce the production of guanylate cyclase. Various disease conditions such as diabetes, atherosclerosis, hypertension and heart disease show a common risk characteristic i.e. high thrombus formation due to increased platelet activity. Researchers believe that platelet hyperactivity may occur due to procoagulant mechanisms as well as loss of the preventive effect of antiaggregants. Additionally, platelet resistance against suppressive effect of insulin and decreased secretion of antiaggregants (including NO and PGI_2_) by endothelium, resulting in loss of platelet control as well as its contact with endothelium [148]. Probably, various mechanisms involved in regulation of platelet activity including platelet–vascular wall contact platelet–agonist relation, platelet–coagulation factor relationship and platelet–platelet interface been suggested to be impaired [148].

In diabetes, several factors including impaired fibrinolysis, increased coagulation, endothelial dysfunction and platelet hyperreactivity, contribute to the prothombic condition that describes patients with T2DM [7, 13, 15, 16, 49, 149, 150]. Platelets are characterized to be hyperactive with increased activation, adhesion and aggregation due to dysregulation of several signaling pathways in T2DM patients [7, 13–16, 151]. Platelets involve in disease pathology by not only triggering thrombus formation but also by releasing oxidative, mitogenic and vasoconstrictive substances that induce the development of local vascular lesions. Furthermore, elevated baseline activation and platelet hyperreactivity in diabetic patients are associated with various metabolic conditions like hyperglycemia, insulin resistance, obesity, dyslipidemia, increased systemic inflammation and oxidative stress [13–16].

### Hyperglycemia and platelet hyperactivity

**Fig. 3:**
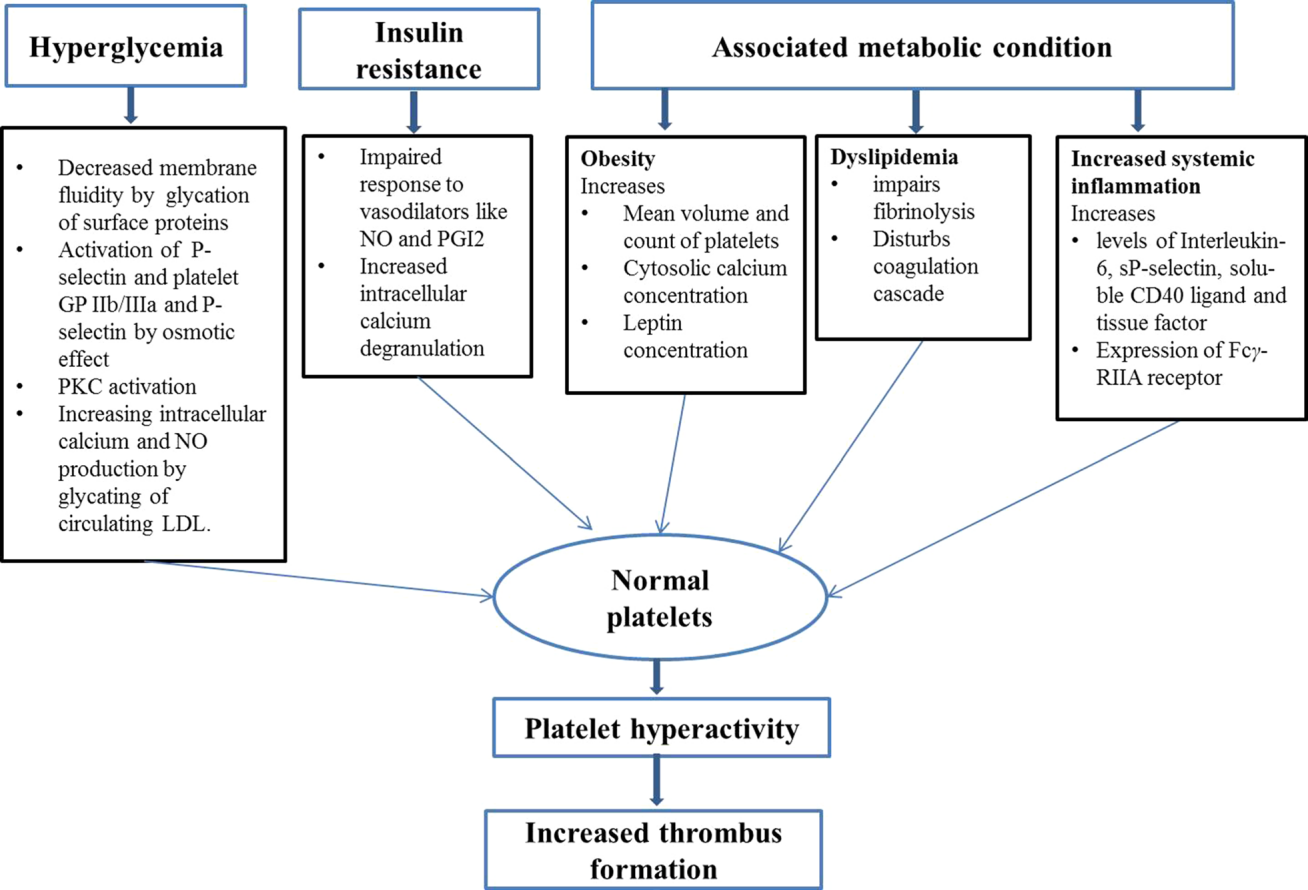
Schematic representation of various biochemical factors responsible for platelet hyperactivity in T2DM

As mentioned already, hyperglycemia plays important role in development of various vascular abnormalities leading to prothrombotic state of diabetic patients [152, 153]. A number of mechanisms are associated with hyperglycemia to increase the platelet reactivity (Fig. 3). The first mechanism is glycation of platelet surface proteins, which increase platelet adhesion by impairing fluidity of membrane [154, 155]. Furthermore, exposure to hyperosmolarity activates platelet GP IIb/IIIa and P-selectin expression, suggesting that hyperglycemia may have direct osmotic effect [156]. Moreover, both chronic and acute hyperglycemia also induces in vivo PKC activation, a transduction pathway that triggers platelet activation [157]. Unlike in healthy subjects, platelets from diabetic patients in vitro also show temporary calcium-sensitive PKC*β* isoenzyme activation under acute hyperglycemia, even without any additional stimuli, demonstrating inherent diabetes-associated impairment of this pathway. In addition, glycation of circulating LDL results in augmented No production and increased intracellular calcium concentration [158]. Specifically, hyperglycemia induces coagulation mechanism by increasing release of prothrombotic molecules like vWF and tissue factor, while inhibits fibrinolysis by raising PAI-1 concentration [159, 160].

Furthermore, platelets are characterized by increased expression of adhesion molecules in T2DM. Platelets of T2DM patients have augmented expression of platelet activation markers (CD31, CD49b, CD62P and CD63) as compared to healthy individuals [161, 162]. A significant reduction in expression of these markers was noticed with improved metabolic control. Moreover, a significant association was observed between glycated hemoglobin (HbA1C) levels and CD62P as well CD63. The above findings suggest that improving glycaemic control can be helpful in reducing platelet adhesion and activation [161, 162]. Occurrence of acute hyperglycemia in healthy subjects, may results into increased platelet activation and reactivity, evidenced by elevated levels of surface (P-selectin and CD40 ligand) as well as soluble markers (sP-selectin) [162–165]. Furthermore, P-selectin expression was found to be associated with fasting glucose and HbA1C, suggesting that platelet activity can be reduced by improved glycemic control in T2DM patients with coronary angioplasty [162].

### Insulin resistance and platelet hyperactivity

**Fig. 4:**
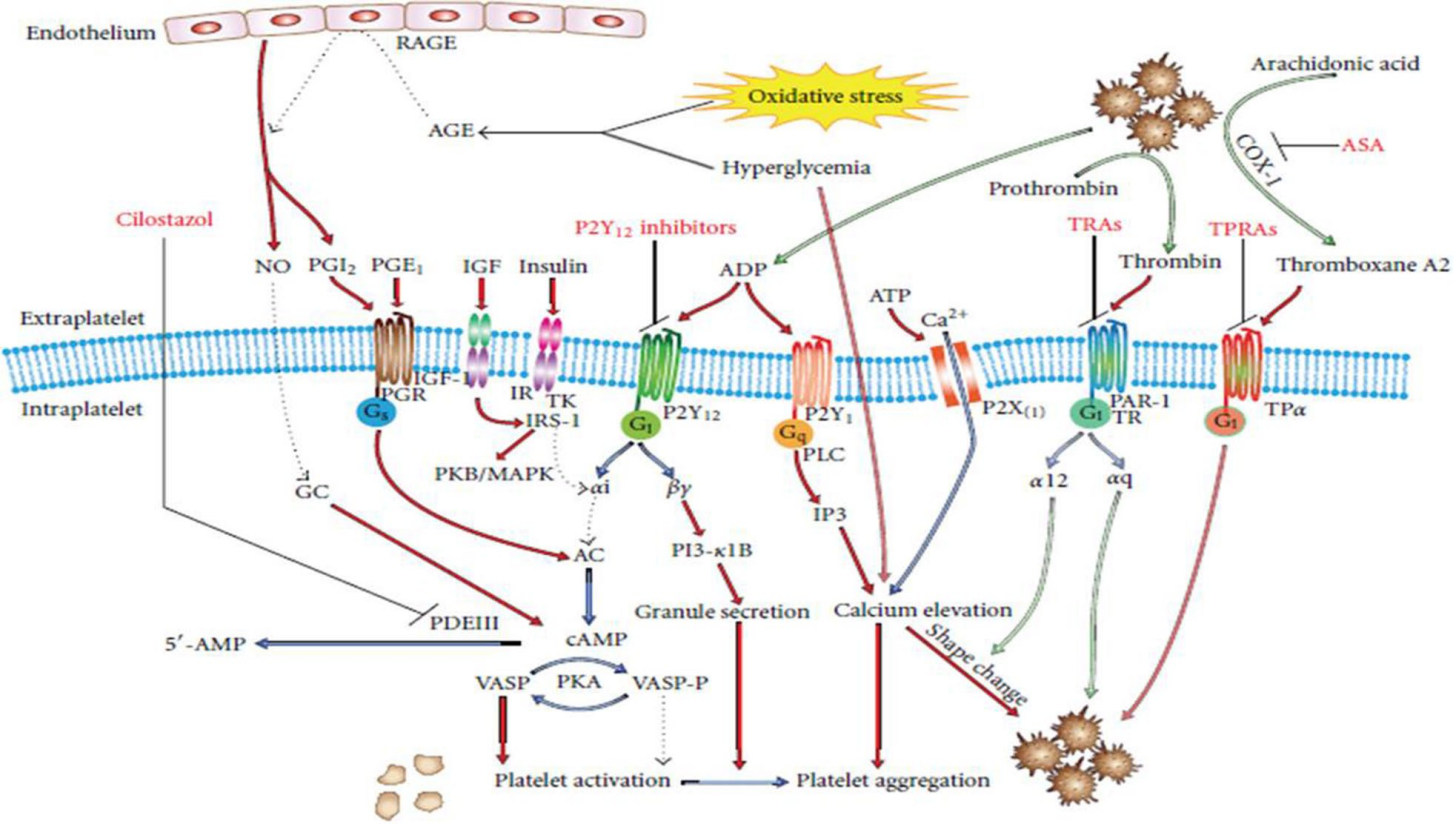
Mechanisms responsible for platelet hyperreactivity in T2DM patient: Elevated cAMP levels leading to platelet inhibition via cAMP-dependent protein kinase (PKA), which disrupt signaling via receptor activation, thromboxane A2 production, MAPK pathway, activation of PKC. The transmembrane G-protein associated receptors involved are prostaglandin, P2Y, P2X, TR, and TP. Both TR and TP present novel drug targets. Antiplatelet drugs are shown in red. *NO* nitric oxide, *AGE* advanced glycation end products, *RAGE* AGE receptors, *PKA/B/C* protein kinase A/B/C, *TK* tyrosine kinase, *PI-3* phosphoinositol-3 kinase, *MAPK* p38 mitogen-activated protein kinase, *GC* guanylate cyclase, *PAR-1* protease activated receptor, *TR* thrombin receptor, *TPα* thromboxane receptor, *TRA* thrombin receptor antagonist, *TPRA* thromboxane receptor antagonist, *ASA* acetylsalicylic acid (aspirin) (Adapted from Kakouros et al. [16])

Impaired insulin action is a prime factor for the development of T2DM [15, 166]. In prediabetes, pancreatic *β*-cells produce compensatory increased insulin to maintain fasting euglycemia. In predisposed individuals, under increased insulin demand, the pancreatic *β*-cells undergo apoptosis, resulting in *β*-cell mass reduction. Eventually, increased insulin in prediabetic condition gradually leads to relative and absolute insulin deficiency. Platelet functions are directly controlled by insulin through a functional insulin receptor (IR) present on platelet surface [167]. The Impact of hyperinsulinemia on platelets is complex and varying between patients with insulin resistance and healthy individuals. In healthy individuals, binding of insulin to IR in vitro leads to translocation of magnesium to platelets, resulting into reduced platelet aggregation and decreased release of proaggregatory agents (e.g. thromboxane B2) [168]. Along with other effects on platelets, insulin induces plasminogen activator secretion and increases expression of PGI_2_. Furthermore, insulin binding to IR located on platelets activates insulin receptor substrate 1 (IRS-1) via tyrosine phosphorylation and mediates its association with G*iα*-subunit [169, 170]. This leads to decreased activity of Gi, which in turn impairs tonic cAMP suppression, raises intraplatelet cAMP levels, reducing signaling of P2Y12 and resulting into decreased platelet activity (Fig. 4). It is also suggested that the relevance of these effects can be restricted by dimerization of subunits of IR and insulin-like growth factor 1 (IGF-1) receptor [171]. Alike IR, IGF-1 receptors are also expressed in platelets [172]. However, binding of IGF-1 to the IGF-1 receptor leads to activation of downstream IR mediators (IRS-1 and IRS-2), resulting in increased platelet reactivity [173]. Moreover, previous in vivo studies involving healthy individuals have proposed that insulin impairs platelet interaction and collagen and also reduces its sensitivity to proaggregants [169, 174].

### Oxidative stress and platelet hyperactivity

As already mentioned, patients with T2DM have been reported to have increased oxidative stress. In these patients, oxidative stress increases intra platelet calcium concentration by platelet activation and facilitating platelet aggregation response [43]. Increased oxidative stress, mainly in uncontrolled T2DM, may leads to platelet hyperactivity by three different ways. Firstly, by increasing production of F2 isoprostane (prostaglandin like compound considered as a marker of oxidative stress), which may enhance platelet response to agonists. Secondly, by reduction activity of eNOS, leading to decreased NO production. The last way is by increasing signaling of platelet receptors [175].

### Other metabolic conditions and platelet hyperactivity

In T2DM patients, other associated metabolic condition like obesity, dyslipidaemia and increased systemic inflammation may also contribute to increase platelet hyperactivity. Obesity is a common feature of patients with T2DM and can induce or exacerbate insulin resistance, which has significant consequences for platelet reactivity [13, 15, 176]. Furthermore, various metabolic abnormalities caused by obesity may leads to platelet hyperactivity. These abnormalities includes increased count and mean volume of platelets, which is associated with platelet reactivity and used as prognostic marker in various atherothrombotic conditions like acute coronary syndrome and stroke [176, 177]. Furthermore, increased cytosolic calcium concentration also enhances platelet reactivity [178]. In addition, high serum leptin concentration also leads to augmented platelet aggregability [179]. Overall, these abnormalities lead to increased platelet adhesion and activation [180, 181]. In a previous report involving subjects with central obesity, it was observed that weight loss induced by diet, reestablished sensitivity for NO and PGI_2_ and decrease platelet activation [182]. Moreover, obese patients were reported to have increased plasma CD40L and elevated levels of derived microparticles, which released in blood on platelet activation [176, 183–185]. Furthermore, subjects with higher BMI were detected to show impaired response to antiplatelet drugs like clopidogrel [186–188]. In obese women, it was observed that weight loss and insulin sensitization by pioglitazone lead to decreased levels of platelet activation markers [180, 183, 185].

T2DM patients are very susceptible to dyslipidaemia, comprised of increased triglycerides, elevated low-density lipoprotein cholesterol (LDL-C) and reduced high-density lipoprotein cholesterol (HDL-C) [13, 15]. Hypertriglyceridemia was reported to increase platelet activation, which is suggested to be mediated by apolipoprotein E, a content of triglyceride rich very low density lipoproteins (vLDL) particles [189–191]. Along with platelet activation, vLDL particles also impair fibrinolysis and disturbs coagulation cascade, thus resulting in atherothrombotic risk [191]. Furthermore, low HDL-C was observed to be associated with endothelial dysfunction in T2DM patients, leading to increased atherosclerosis [192]. Furthermore, it has been reported that in hypercholesterolemia patients, intravenous infusion of HDL reconstitution stabilizes endothelial function by increasing NO bioavailability in these patients [193]. In another report, it was observed that reconstituted HDL decrease platelet aggregation by inducing cholesterol efflux from platelets in diabetic patients [194].

Furthermore, the association of T2DM with increased systemic inflammation is well known. In T2DM, increased levels of inflammatory, coagulation and platelet activation markers (interleukin-6, sP-selectin, soluble CD40 ligand) and tissue factor were observed in comparison with healthy controls [195]. Furthermore, it was suggested that in diabetic patients inflammation increases expression of Fc*γ*-RIIA receptors, which induces increased platelet activation in response to collagen [196, 197].

### Biomarkers of platelet hyperactivity in T2DM

Various important markers of platelet activation includes sP-selectin, platelet factor 4 (PF4), β-thromboglubulin (β-TG), glycoprotein V (CD42d), thromboxane B2 (TXB_2_) and thrombospondin-1. P-selectin (CD62P) is a carbohydrate-binding lectins stored in platelets as well as endothelial cells. It is well known as a marker of platelet activation [198, 199]. Elevated levels of P-selectin are already reported in T1DM and T2DM patients without vascular complications [199–201]. Furthermore, in patients with peripheral artery disease (PAD), arterial hypertension CAD increased levels of P-selectin in plasma were associated with levels of β-TG but not with vWF and TM (markers of endothelium activation) [202–204]. Pf4, a basic protein stored in platelet granules, is characterized with both anticoagulant and procoagulant properties. It also impairs migration and proliferation of endothelial cells [205]. Other important platelet protein β-TG is having 50% structural similarity with PF4 and act as a leukocyte chemoattractant. The estimation of β-TG in patients with normal renal function is considered as gold standard platelet activation detection. Increased levels of both of PF4 and β-TG were suggested as indicator of platelets degranulation in patients with both T1DM and T2DM without vascular complications [205]. Glycoprotein V is also expressed on platelets and megakaryocytes, and it binds to the complex GPIb/GPIX non-covalently and forms a receptor for both vWF and thrombin [206]. The other factor TXB2 is an inactive metabolite/product of thromboxane A2. As release of TXA_2_ increased in platelets, TXB2 levels were also elevated in platelets, liver, kidneys and lungs in correlation with increased β-TG and PF4 levels [207]. The other important marker thrombospondin-1 (TSP-1) is multifunctional glycoprotein mainly expressed on various types of cells including vascular smooth muscle cells, platelets and renal cells. After release of thrombospondin from platelets, TSP-1 binds to the surface of activated platelets. In a previous report, levels of TSP-1 were suggested to confirm renal damage and vascular impairment in patients with diabetic nephropathy [208].

Different platelet markers can be studied by using enzyme linked immune sorbent assay (ELISA) and Western blot. A standardized method like flow cytometry is widely used for estimation of platelet function. It has various advantages over other techniques as it requires small blood volume and is independent of platelet count [166, 209, 210]. It also permits expression analysis of various platelet activation markers as well as platelet receptors on distinct platelets. Furthermore, flow cytometry allows the quantification analysis of the associates that link platelet to the other blood cells [211–215].

Mean platelet volume (MPV), an important marker of platelet activation, gives idea about platelet size and activation of larger platelets represent more activity [216]. T2DM patients have been reported to have significantly higher MPV than controls, which is contributed to increased cytoplasmic alpha granule content, indicating impairment platelet-megakaryocyte system [216–218]. Hence, platelets can be an important entity to estimate vascular risk in T2DM [197, 198]. Furthermore, studies have observed independent association of MPV with diabetes and increased MPV indicates early diabetic thrombocytopathy in both type of DM without vascular complications [205, 219].

### Pharmacological influences on platelet hyperactivity and its biomarkers

To reduce the vascular risk in the T2DM patients, studies have reported various therapeutic targets for platelet hyperactivity. As already mentioned above, platelet derived microparticles (PMP) are shown to be involved in the increased development of atherosclerotic plaque and formation of arterial thrombosis in diabetic patients. Undeniably, T2DM patients have platelets that are described by a hyperactive phenotype with increased activation, adhesion and aggregation that may attributable to development of atherothrombotic complications in these patients [220–222]. Therefore, elevated levels of specific microparticles can be used as important predictor of vascular injury and cardiovascular outcome, especially in patients with T2DM [223–226]. Furthermore, studies have indicated that PMP may transport platelet miRNA to a specific site in cardiovascular system, which may be used as novel biomarkers to improve diagnostics and treatment of vascular complications in diabetic patients [227]. Platelet-derived miRNAs i.e. miR-223 and miR-126 have been shown to have highest association with CVD. miR-223 controls erythrocyte membrane protein band 4.1 like 3 (*EPB41L3*) gene, known to be associated with atherosclerosis. However, miR-126 regulates a VCAM-1 gene, known to be linked with endothelial dysfunction and atherosclerosis, indicating important role of platelet-derived miRNAs in regulation of key genes associated with atherosclerotic vascular complications [228, 229]. In another study, *N*-acetylcysteine was reported to provide protection against the risk of stroke by altering both systemic as well as vascular prothrombotic responses and correcting levels of antioxidants in T2DM patients [230].

Various antiplatelet and anticoagulant drugs have been suggested as novel therapeutic strategies to improve platelet function in T2DM [231–237]. Acetylsalicylic acid (aspirin), a cyclooxygenase (COX) inhibitor, has been known to have anti-inflammatory and antithrombotic properties from past several decades [233, 234]. In addition, aspirin is also used as a key molecule in antiplatelet treatment to decrease mortality due to myocardial infarction or stroke. However, in recent studies, low-dose aspirin was found to be associated with increased risk for gastrointestinal bleeding in T2DM patients in a primary prevention setting [238]. In addition, for long-term treatment or prevention of thromboembolic complications, use of oral anticoagulants is recommended [231]. From the past decades, vitamin K antagonists have been shown to have anticoagulant properties. The most important vitamin K antagonists that are currently in use are dicoumarol warfarin, phenprocoumon, acenocoumarol and rivaroxaban [239–242]. However, risk of a recurrent event among patients with venous thromboembolism has shown to be decreased with rivaroxaban at either a treatment dose (20 mg) or a prophylactic dose (10 mg) than with aspirin, without a significant increase in bleeding rates [243]. Furthermore, P2Y12 receptor antagonists such as clopidogrel, ticagrelor, prasugrel and cangrelor have been shown to chemically block the adenosine diphosphate (ADP) receptors (P2Y12) [231]. The overall effect on platelet includes reduction in platelet aggregation, conformational changes, emergence of pseudopodia and interaction with other cellular or plasma components involved in coagulation [244]. Furthermore, Glycoprotein IIb/IIIa inhibitors such as Abciximab, tirofiban and eptifibatide, inhibit the fibrinogen adhesion to activated platelet, blocking the formation of interplatelet bridges [231, 245, 246]. Furthermore, various anticoagulants have been shown to prevent the initiation as well as progression of coagulation and fibrin-clot formation/propagation [237, 247, 248]. These drugs are being used for the prevention or treatment of atrial fibrillation and venous thromboembolism. These anticoagulants include unfractionated heparin, low molecular weight heparin. The recently introduced parenteral anti-factor Xa agents (fondaparinux) have widely replaced the unfractionated heparin [231].

## Conclusion

Hyperglycemia and insulin resistance, the cardinal characteristics of T2DM, are responsible for affecting normal endothelial and platelet function, which are linked with the development of prothrombotic state in T2DM patients. This metabolic milieu increases the vascular risk in these patients, accounting for high mortality and morbidity rate observed in T2DM. Thus, understanding of mechanisms of endothelial and platelet dysfunction is emerging as a major issue in prevention and management of vascular complications in T2DM.

## Abbreviations

ADMA: asymmetrical dimethylarginine
AGEs: advanced glycation end products
β-TG: β-thromboglubulin
CAD: coronary artery disease
CD42d: glycoprotein v
CRP: c-reactive protein
DM: diabetes mellitus
ELISA: enzyme linked immune sorbent assay
eNOS: endothelial no synthase
EPCs: endothelial progenitor cells
ET-1: endothelin-1
FFAs: free fatty acids
FMD: flow-mediated dilatation
GLP-1: glucagon-like peptide-1
GP: surface glycoproteins
HbA1C: glycated hemoglobin
HDL-C: high-density lipoprotein cholesterol
ICAM: inter-cellular adhesion molecule
IDF: International Diabetes Federation
IGF-1: insulin-like growth factor 1
IL-6: interleukin-6
IR: insulin receptor
IRS: insulin resistance substrate
JNK: C-jun n-terminal kinase
LDL-C: low-density lipoprotein cholesterol
MAPK: mitogen activated protein kinase
miRs: micrornas
MPV: mean platelet volume
NF-κB: nuclear factor-κb
NO: nitric oxide
oxLDL: oxidized low-density lipoprotein
PAD: peripheral artery disease
PAI-1: plasminogen activator inhibitor-1
PARP: poly (adp-ribose) polymerase
PET: positron emission tomography
PF4: platelet factor 4
PGI_2_: prostacyclin
PI3K: phosphatidylinositol-3-kinase
PKC: protein kinase c
PMP: platelet derived microparticles
RAGE: age receptors
RNS: reactive nitrogen species
ROS: reactive oxidative species
T2DM: type 2 diabetes mellitus
TNF-α: tumor necrosis factor-α
TXB_2_: thromboxane b2
VCAM: vascular adhesion molecule
VLDL: very low-density lipoprotein
VSMC: vascular smooth muscle cells
vWV: von willebrand factor
WHO: World Health Organization
Wnt: wingless-type family member
